# An Address on the Present State of Therapeutics

**Published:** 1868

**Authors:** Thomas Watson


### ARTICLE VI.

*An Address on the Present State of Therapeutics.—Delivered at the Opening Meeting of the Clinical Society of London.* By Sir Thomas Watson, Bart., M.D.

I am very sensible of the great honor which you have done me in electing me the first President of the Clinical Society. Reluctant as I not unnaturally am to assume at my time of life any fresh duties or obligations, I yet must confess that I have extreme satisfaction and pleasure in accepting at your hands this new office; for the Society which we are founding to-night seems to me well calculated gradually to bring about that which, in my judgment, is the thing most needful at present amongst us. I mean more exactness of knowledge, and, therefore, more direct and intelligent purpose, and more successful aim, in what is really the end and object of all our labors—the application of remedies for the cure or relief of disease. Certainly the greatest gap in the science of medicine is to be found in its final and supreme stage—the stage of therapeutics. The anatomy of the human body is sufficiently well known. Its material pathology, also, under the auspices especially of a sister Society, has been, I will not say completely, yet very amply and fruitfully ransacked, by the diligent scrutiny and study of the dismal but instructive revelations of the dead-house. I say its *material* pathology, for the condition of doctrinal pathology must necessarily partake of whatever imperfection may be found in the correlative science of physiology. Again, we have attained to a great degree of certainty in the detection and discrimination of disease in the living body. We know tolerably well *what* it is that we have to deal with, but we do not know so well, nor anything like so well, *how* to deal with it. This is more true, no doubt, in the province of the physician than in that of the surgeon, but it is lamentably true in both provinces. We want to learn distinctly what is the action of drugs, and of other outward influences, upon the bodily organs and functions,—for every one now-a-days. I suppose, acknowledges that it is only by controlling or directing the natural forces of the body that we can reasonably hope to govern or guide its diseased actions. To me it has been a life-long wonder how vaguely, how ignorantly, how rashly drugs are often prescribed. We try this, and, not succeeding, we try that, and baffled again, we try something else; and it is fortunate if we do no harm in these our tryings. Now, this random and hap-hazard practice, whenever and by whomsoever adopted, is both dangerous in itself, and discreditable to medicine as a science. Our profession is continually fluctuating on a sea of doubts about questions of the gravest importance. Of this the evidence is plentiful and constant. Let me substantiate what I am now saying by one or two glaring instances. The old, and, as might have been hoped, obsolete controversy between the Cullenian and the Brunonian schools has been revived in all its former extravagance within our own time. Many of us can recollect the period when bloodletting was reckoned the *summum remedium* against at least all forms of inflammatory disorder—which were to be starved out also by the strict enforcement of what was called the antiphlogistic regimen. Now, there are, I believe, many who yet hold that to deprive a patient of an ounce of his blood is to sap his strength and to aggravate his danger, and that for all ailments brandy is the grand and easy panacea. One generation extols mercury as the sole and unfailing remedy for syphilis; the next attributes all the worst evils that follow in the train of that hateful disorder to the very mineral which had been administered for its cure. Even now, at this present time, a hot contention, of most weighty import, fills the air around us, upon the question whether, when cholera is present in the community, we should treat the diarrhœa, presumed to be the prelude or the commencement of cholera, by opium and astringents to check the discharges from the bowels, or by castor oil to promote them.

I say this uncertainty, this unseemly variation and instability of opinions, is a standing reproach to the calling we profess. It has shaken the faith of many men, of men both able and thoughtful, and driven them to ask themselves whether any kind of medication, other than the *vis medicatrix naturæ*, is of any real efficacy or value. Well, this is one of the questions which it will be competent for the Clinical Society to settle.

In order to clear the ground for correct observation, and in order to the avoidance of fallacies in observing, it is most desirable, when it can be done without harm or known hazard to the sick, to learn respecting all distinct and recognized forms of disease, what would be their course, what their tendencies, what their results, if left to themselves and subjected to no kind of remedial treatment whatever; to ascertain, in a word, what it has become the fashion to speak of as the *natural history* of disease. For this purpose, again, the Clinical Society may be expected to furnish help. Truly, there are diseases in which it seems to be our main business to stand by and look on; to see that Nature has fair play; that the patient has the requisite advantages of rest, and warmth, and pure air, appropriate food, and no more; to watch his recovery, not to attempt his cure. Probably all the speficic fevers that run a definite course are of this kind. Medicine needs to step in only to redress some untoward deviation from that regular course, or to facilitate and fortify the natural recuperative efforts. But there is a legion of other disorders for which rest, and warmth, and pure atmosphere, and well adjusted diet, are *not* sufficient. There *are* cures as well as recoveries; and there *are* remedies that are equal to the cure. Still, of therapeutics as a trustworthy science, it is certain that we have as yet only the expectation. The influence of drugs upon the bodily conditions of health and disease is, indeed, most real and most precious to us. And some of them, in our contests with disease, we have learned to wield with much confidence and success.—Who can doubt the efficacy of opium and of anæsthetic vapors in blunting the sensibilities of the body, and so quelling pain? No one questions the marvellous power of quinine to stop malarious fevers and other periodic complaints, or of the iodide of potassium to eliminate from the body, apparently by first dissolving them, certain poisonous or hurtful elements. The rough yet sanative effects of emetic and purgative drugs are notorious to all. But there is a host of other known or reputed remedial substances—to say nothing of a further host, no doubt, hitherto unthought of and unessayed—about which our practical knowledge is very loose, and imperfect, or even misleading. Concerning the peculiar virtue and specific agency of each and all of these, present and to come, we want sound and multiplied experience. There is no other way. The required knowledge must needs be gathered empirically, and by many hands. And while there are many drugs and medicaments yet unproven, there are also many shapes of disease of which the true nature and origin are still disputed or doubtful. Of all these matters will this Clinical Society—if I rightly apprehend its scope and purpose—take cognisance. Full and faithful descriptions brought before it, by competent and accurate observers, of the symptoms, circumstances, and progress of disease in the living body, and of its behavior under treatment by medicines prescribed with singleness and simplicity, and a definite aim and object, or sometimes, it may be, of its behavior under no treatment at all—authentic reports of trials with medicinal substances upon the healthy human body,—contributions of this order, multiplied in number, compared together, contrasted, sifted, and discussed by a variety of keen and instructed minds, of minds sceptical in the best and true sense of that word,—must lead at length, tardily perhaps, but surely, to a better ascertainment of the rules—peradventure, to the discovery even of the laws—by which our practice should be guided; and so bring up the therapeutic and crowning department of medicine to a nearer level with those other parts which are stricly ministerial and subservient to this. And I think I do not entertain an extravagant expectation of the results of the formation of this Society when I express my belief that, if wisely and strictly managed, it will hereafter be spoken of as the starting-point of a vast and solid improvement in that which is our special office in the world—the scientific and intelligent exercise of the divine art of healing.

